# A methodology for multivariate phenotype-based genome-wide association studies to mine pleiotropic genes

**DOI:** 10.1186/1752-0509-5-S2-S13

**Published:** 2011-12-14

**Authors:** Sung Hee Park, Ji Young Lee, Sangsoo Kim

**Affiliations:** 1 Department of Bioinformatics and Life Science, Soongsil University, Seoul, R.O. KOREA

## Abstract

**Background:**

Current Genome-Wide Association Studies (GWAS) are performed in a single trait framework without considering genetic correlations between important disease traits. Hence, the GWAS have limitations in discovering genetic risk factors affecting pleiotropic effects.

**Results:**

This work reports a novel data mining approach to discover patterns of multiple phenotypic associations over 52 anthropometric and biochemical traits in KARE and a new analytical scheme for GWAS of multivariate phenotypes defined by the discovered patterns. This methodology applied to the GWAS for multivariate phenotype *highLDLhighTG* derived from the predicted patterns of the phenotypic associations. The patterns of the phenotypic associations were informative to draw relations between plasma lipid levels with bone mineral density and a cluster of common traits (Obesity, hypertension, insulin resistance) related to Metabolic Syndrome (MS). A total of 15 SNPs in six genes (*PAK7*, *C20orf103*, *NRIP1*, *BCL2*, *TRPM3*, and *NAV1*) were identified for significant associations with *highLDLhighTG*. Noteworthy findings were that the significant associations included a mis-sense mutation (*PAK7*:R335P), a frame shift mutation (*C20orf103*) and SNPs in splicing sites (*TRPM3*).

**Conclusions:**

The six genes corresponded to rat and mouse quantitative trait loci (QTLs) that had shown associations with the common traits such as the well characterized MS and even tumor susceptibility. Our findings suggest that the six genes may play important roles in the pleiotropic effects on lipid metabolism and the MS, which increase the risk of Type 2 Diabetes and cardiovascular disease. The use of the multivariate phenotypes can be advantageous in identifying genetic risk factors, accounting for the pleiotropic effects when the multivariate phenotypes have a common etiological pathway.

## **Background**

Genome-wide association studies (GWAS) have broadened our knowledge on architectures of disease susceptible loci for many common disease of public health importance. A general approach for GWAS follows a strategy to investigate the correlations between single genetic variants and single traits within a univariate framework. The GWAS have not considered complicated genetic nature such as pleiotropy that occurs due to potential genetic correlation between different traits [1,2]. Thus, it tends to be restricted to identify pleiotropic genes that situated at common etiologic pathways of correlated human diseases.

Patterns of pleiotropic effects have been observed more with an increasing number of variants identified through GWAS [3]. For instances, Winkler and colleagues identified a variant of TCF2 (Transcription Factor 2) associated with T2D [4], while a different variant in the same gene was associated with an increased risk of prostate cancer [5]. These two studies indicate that the risk allele for prostate cancer protects from T2D with an odds ratio of 0.91. In addition, two studies [3] showed that the same variant in GDF5 associated with greater height also was associated with reduced risk of osteoarthritis [6,7].

As we have mentioned the examples above, previous work has shown that ignoring pleiotropic effects may cause imprecise phenotype definition of heterogeneous samples or even spurious associations. A bias in sampling cases and controls characterizing single traits might be propagated since the sampling errors tend to be correlated if the single traits were correlated. This may confound the interpretation of results. Although any loss of power occurred by selection of samples can be recovered by increasing the sample size, the sample size of the GWAS has cost constraints. With large sample sizes of several thousand cases and controls there has been usually limited study power to detect alleles of modest effect sizes (e.g., an odds ratio of 1.20) [8].

In this regard, incorporation of the multiple phenotypes to the GWAS can be an alternative way to unravel missing heritability in the GWAS and to find pleiotropic genes. Even though the GWAS of multivariate phenotypes are known to enhance the power of the GWAS such an approach has not been well established.

To perform multiple phenotypes based GWAS, application of traditional GWAS approaches has suffered penalties from multiple testing problems caused by testing multiple genome-wide scans of single traits separately. This may diminish the power of GWAS due to elevating heterogeneity and bias in samples. Statistically classic multivariate methods have been applied to GWAS of multivariate phenotypes to tackle in an effective manner. Such methods are likelihood-based mixed effects model (LME) [9,10] and generalized estimating equations (GEE) methods. Liu et al. suggested an extension of the GEE to test association analysis for a mixture of continuous and binary traits [11]. Their work manifested statistical power of bivariate association analysis with two continuous traits, i.e. obesity and osteoporosis. Their method is limited to bivariate traits and applicable to independent samples.

O’Brien model [12] and its extension [13], which suggested the integration of results from association tests of single traits of a multivariate phenotype, can work well for a homogeneous mean among individual tests of single traits but not for heterogeneous ones. To overcome this limitation, Yang et al. [2] improved O’Brien method by use of a sample splitting method and a cross validation method as a screening tool for detecting pleiotropic effects. Previous work has contributed to addressing association tests for multivariate phenotypes. However, there is still no standard method to be free from multiple test problems and be accepted for multivariate phenotypes [11].

Much work have not investigated what types of single traits can be correlated to induce multivariate phenotypes. In this context, we aimed to discover novel multivariate phenotypes from large scale epidemiological data by a data mining approach and develop a scheme to GWAS of multivariate phenotypes. In our previous work [14], we reported the discovery of multivariate phenotypes by applying association rule mining over 52 anthropometric and biochemical traits in Korea Association Resource (KARE)[15] population. We showed an analytical scheme for GWAS of the multivariate phenotype *lowLDLhighTG*, which means a negative relation between low levels of LDL and high levels of TG. Our preliminary results revealed that effect sizes (odds ratios=1.44-2.38) of genetic loci associated with the multivariate phenotype were higher than genetic loci identified in the initial GWAS, while their p-values were less significant than those in the initial GWAS. Those loci cannot be detected within a single trait based framework.

Here, we present a more sophisticated scheme for refining association rules to extract patterns of phenotypic associations and to visualize them graphically. As a case study, we describe the results of GWAS for multivariate phenotype *highLDLhighTG* combining elevated low density lipoprotein cholesterol (LDL-C) levels and elevated triglyceride (TG) levels, which have an important clinical implication in metabolic syndrome (MS).

An association rule which expresses patterns of multivariate phenotypes encoding partial correlations between single traits specifies quantitative descriptions of the single traits. Association rules can provide explicit boundaries of the single traits of multivariate phenotypes for optimal selection of both cases and controls. This work contributes a methodology for exploration in GWAS analysis of multiple phenotype *highLDLhighTG*.

## **Methods**

### Data

We investigated 350K variants in 8,512 individuals in Korea Association Resource (KARE) for performing GWAS of multivariate phenotypes and mining patterns of them. Additional details of quality-control and imputation procedures have been reported in [15]. Gene annotations refer to the human genome build hg18. Of 8,842 individuals in KARE, 1,853 having missing values found in at least one of traits were removed. The individuals were described by a total of 52 traits (see Additional file 1), of which six traits (CRP, AST, ALT, r_gtp, homa, creatin) extremely distributed were removed. The resulting 6,998 individuals with 46 traits were employed in association rule mining.

### Association rule mining

The problem of discovery of multivariate phenotypes from a set of single traits is transformed into finding frequent patterns of associations of single traits. In our approach we employed Association Rule Mining (ARM) [16] to discover the patterns of phenotypic associations expressed as association rules. We have previously shown [14,17] that association rules detected by ARM are informative and quantitative and have benefits to interpret their meaning. Association rules have the form R: X → Y [c, s], where X (the Left-hand side or LHS) and Y (the Right-hand side or RHS) are the body and the head of a rule, respectively. c and s stand for confidence and support to measure accuracy of rules. X and Y are disjoint predicates (X ∩ Y = Ø). Each X and Y consists of a conjunction of distinct predicates which describe values of single traits. An association rule expresses association of single traits X and Y and can be derived to a multivariate phenotype.

The strength of the association rules can be measured in terms of their support (s) and confidence (c). The support of a rule (X → Y) is the probability that cases in a database contain both X and Y. The confidence of a rule is the probability that cases contain X can also contain Y. Note that the head of a rule, Y, is restricted to be one of the single traits which we are interested in and X is a set of traits partially correlated with Y. Interesting rules are manually extracted.

### Discovery of multivariate phenotypes

#### Association rule generation and post filtering the rules

We used 10g Oracle Data Miner (ODM) which implemented the APRIORI algorithm [16] to compute association rules. We set a minimum support and a minimum confidence of 1% and 10%, respectively, to detect rare patterns representing disease predisposing cases more rather common features appeared in normal cases.

If we set threshold too high for support (e.g., 20 %), there may miss many interesting patterns involving the low support with high confidence. Such low support traits may correspond to rare associations of sing traits, but whose patterns are still interesting to know. Most patterns mined characterize common correlations between traits, which are already well known. Domain experts such as biologists may have different interesting traits. Our strategy to find interesting rules is that we set low support threshold, generate as many rules and filter them by user interestingness measurements and interesting traits as user constraints. High confidence rules can misinform and do not consider the support of the traits appearing in the rule head. One way to resolve this problem is to apply a metric known as lift [18]:

*Lift*=*P*(*X*,*Y*)/*P*(*X*)×*P*(*Y*)

which calculates the ratio between the rule’s confidence and support of the single trait *Y* in the rule consequence. Lift was originally called interest which measures how many times more often *X* and *Y* occur together than expected if they are statistically independent.

The following example shows rule form constraints to find interesting association rules containing single traits such as high levels of TG (TG5) and low levels of LDL(LDL1):

R1: ((TG5⊂LHS) ∨ (TG5⊂RHS)) ∧ (min_conf ≥ 0.7, lift ≥ 1, min_sup ≥ 0.025),

R2: ((LDL1⊂LHS) ∨ (LDL1⊂RHS)) ∧ (min_conf ≥ 0.7, lift ≥ 1, min_sup ≥ 0.025).

#### Visualization of phenotypic association

We employed graph-based techniques to visualize interesting association rules using *igraph* package on R 2.12.0. A set of interest association rules represented with an undirected edge weighted graph where vertices represent traits and edges indicate relationships (i.e. associations) between other traits in rules. Vertex size is proportional to degrees of vertices.

### A scheme of association analysis

#### An algorithm for association test

A multivariate phenotype(y) is defined as bivariate traits (y_i_, y_j_) which can be a mixture of continuous and nominal traits. The multivariate phenotype can be a conjunction of predicates of single traits, as expressed one or more association rules.

y = y_i_ + y_j_

Association analysis for multivariate phenotype(y) is performed by following tasks:

(1) Test a genome-wide scan for a multivariate phenotype (y): GWAS(y)

(2) Test genome-wide scans for singleton traits: GWAS(y_i_), GWAS(y_j_)

(3) Calculate mOR_p_^s^ where minus log odds ratio for p-values of SNPs obtained from an association test of a multivariate phenotype(y) over those from a single trait (y_i_) as follows:

(4) Prune SNPs by filtering strategies (see section strategy for pruning SNPs)

(5) Repeat tasks 2~4 for other single trait (y_j_)

(6) A final set of SNPs is generated by the intersection of two sets of SNPs survived from filtering steps which prune SNPs more likely to affect either of single traits.

SNPs identified for an association test of a multiple phenotype may include genetic variants more likely affecting each of single traits which should be excluded. Tasks 1 to 4 are subject to filtering steps to identify genetic variants influencing the multivariate phenotype y while the genetic variants much more likely to affect single traits (y_i_ and y_j_) are excluded by mOR_p_^S^ (vide infra). The mOR_p_^S^ measures the strength of association of a multivariate phenotype against those of single traits.

#### Strategy for pruning SNPs

From a list of results of the association test for a multivariate phenotype, false positives of SNPs are pruned by the following conditions and significant ones remain:

(1) P-values for an association test of multivariate phenotype ≤ 5×10^-4^

(2) mOR_P_^yi^ ≥ 1 and mOR_p_^yj^ ≥ 1

(3) P-value of an indexed SNP ≤ 10^-5^ and p-values of clumped SNPs ≤ 10^-4^ in order to evaluate formation of LD.

There have been no generalized method to deal with the multivariate phenotypes and the cut-off p-value for a significant association in GWAS is not well defined. The cut-off p-value for the multivariate phenotype is set to be less stringent than usual GWAS which mostly set a Bonferroni corrected p-value of 0.05 (P ≤ 1.43×10^-7^ which is not corrected) since we consider a small sample size for cases and controls due to combination of phenotypes. mOR_P_^S^ ≥1 means that it is 10 times as likely that SNPs are associated with a multivariate phenotype against a single one.

### Application to GWAS of lipid levels

We applied to multivariate phenotype based GWAS to relations between plasma lipid levels for an in-depth study. Positive relations between TG and LDL-C can be specified as multivariate phenotype *highLDLhighTG*, which indicates high LDL-C of ≥ 130 mg/dl and high TG of ≥ 180mg/dl extracted from 359 rules. Ranges of TG and LDL-C levels for GWAS are adjusted for those meeting the guidelines used in clinical researches.

An association test of *highLDLhighTG* seeks to identify single shared loci affecting both high levels of LDL-C and TG while excluding ones affecting either of the single traits LDL-C and TG. 545 cases and 680 controls (Table 1 and see Additional file 2 for single traits) are selected for GWAS of multivariate phenotype *highLDLhighTG*. SNPs influencing on either one of TG or LDL-C traits were pruned from a list of significant SNPs associated with *highLDLhighTG*. Case-control based association scans and LD clumping were performed by PLINK [19].

**Table 1:** Descriptive summary of samples

highLDL highTG	Control (LDL ≤ 100 and TG ≤ 100, n=681)				Case ( LDL ≥ 130 and TG ≥ 180, n=545)			
Area/sex n=individual	Ansung n=381	Ansan n=300	Male n=233	Female n=458	Ansung n=205	Ansan n=340	Male n=288	Female n=257
AGE	52.6 ± 9.45	45.7 ± 5.88	54.1 ± 9.34	47.3 ± 7.56	56.6 ± 8.20	50.1 ± 8.19	49.6 ± 7.99	55.8 ± 8.45
BMI	22.7 ± 3.06	23.2 ± 2.80	21.8 ± 2.71	23.4 ± 2.94	26.0 ± 2.84	25.9 ± 2.71	25.5 ± 2.58	26.4 ± 2.89
WHR	0.88 ± 0.06	0.79 ± 0.07	0.87 ± 0.06	0.83 ± 0.08	0.95 ± 0.05	0.87 ± 0.05	0.90 ± 0.05	0.90 ± 0.08
TCHL	148 ± 18.3	151 ± 15.61	149 ± 17.8	150 ± 16.9	240 ± 22.3	243 ± 24.6	242 ± 22.8	242 ± 24.9
HDL	50.3 ± 10.5	50.0 ± 10.5	51.0 ± 12.0	49.8 ± 9.70	42.4 ± 8.56	42.5 ± 7.66	42.2 ± 7.55	43.0 ± 8.48
**LDL**	81.8 ± 14.8	85.1 ± 11.7	81.6 ± 14.5	84.1 ± 13.1	150 ± 16.8	155 ± 20.9	152 ± 18.6	153 ± 20.7
**TG**	81.4 ± 13.4	81.0 ± 12.4	80.9 ± 14.5	81.4 ± 12.2	238 ± 51.7	231 ± 49.0	237 ± 52.3	230 ± 47.3

**highLDL**	Control (LDL ≤ 100, n=2215)				Case (LDL ≥ 130, n=2271)			

Area/sex n=individual	Ansung n=1403	Ansan n=812	Male n=1024	Female n=1191	Ansung n=919	Ansan n=1352	Male n=1044	Female n=1227
**LDL**	79.6 ± 16.6	84.1 ± 12.4	79.2 ± 17.3	83.0 ± 13.2	151 ± 17.9	155 ± 20.9	154 ± 19.5	153 ± 20.1
**TG**	157 ± 78.5	148 ± 78.0	169 ± 82.2	140 ± 72.5	146 ± 61.8	147 ± 61.3	152 ± 65.3	142 ± 57.6

**highTG**	Control (TG ≤ 100, n=1914)				Case (TG ≥ 180, n=1779)			

Area/sex n=individual	Ansung n=920	Ansan n=994	Male n=700	Female n=1214	Ansung n=936	Ansan n=843	Male n=969	Female n=810
**LDL**	109 ± 29.7	118 ± 30.0	116 ± 31.9	112 ± 29.1	104 ± 33.7	122 ± 33.8	111 ± 35.8	115 ± 33.8
**TG**	81.4 ± 13.6	80.4 ± 13.5	80.0 ± 14.8	81.5 ± 12.7	248 ± 55.5	241 ± 53.6	248 ± 56.1	240 ± 52.6

## **Results and discussion**

### Multiple phenotypic associations

Out of a total 3,792,566 rules mined, 765,318 rules of which lift ≥ 1 and confidence ≥ 0.5 were retained. 136,551 rules encoded TG and LDL levels. Of 19,837 rules related to high TG levels, 191 interesting rules represent low LDL-C and high TG in contrast to 509 rules that manifested high TG and high LDL-C levels. Table 2 denotes the representative association rules (see Additional file 3) and interpretation of rules refer to the previous work [14].

**Table 2:** Representative association rules

Rule #	Rule body		Rule head	Supp	Conf	Lift
	Rules encoding highTG levels					
1	LDL5, BMI5, TG5, TCHL5	→	NONHDL5	0.0157	1.0000	5.1732
2	GLU1205, TCHL5, LDL5, TG5	→	NONHDL5	0.0136	1.0000	5.1732
3	LDL5, TG5, TCHL5, GLU05	→	NONHDL5	0.0132	1.0000	5.1732
4	TG5, PLAT5, LDL5, NONHDL5	→	TCHL5	0.0127	1.0000	5.1465
5	GLU605, TG5, TCHL5, LDL5	→	NONHDL5	0.0126	1.0000	5.1732
6	TG5, LDL5, TCHL5, DS1	→	NONHDL5	0.0122	1.0000	5.1732
7	LDL5, INS1205, TCHL5, TG5	→	NONHDL5	0.0119	1.0000	5.1732
8	DBP5, TG5, LDL5, TCHL5	→	NONHDL5	0.0119	1.0000	5.1732
9	INS605, TCHL5, TG5, LDL5	→	NONHDL5	0.0116	1.0000	5.1732
10	TG5, TCHL5, INS05, LDL5	→	NONHDL5	0.0107	1.0000	5.1732
11	TG5, LDL5, SBP4, TCHL5	→	NONHDL5	0.0107	1.0000	5.1732
12	TG5, TCHL5, WHR5, LDL5	→	NONHDL5	0.0107	1.0000	5.1732
13	T_HDL5, NONHDL5, LDL4, HDL2	→	TG5	0.0102	0.8875	4.1105
14	TCHL2, LDL1, NONHDL2, PH1	→	TG5	0.0100	0.8333	3.8595
	Rules encoding highLDL levels					
15	TCHL5, NONHDL5, GLU605	→	LDL5	0.0405	0.8324	4.2651
16	DS1, NONHDL5, GLU605	→	LDL5	0.0155	0.8182	4.1924
17	NONHDL5, SONA4, TCHL5	→	LDL5	0.0242	0.8450	4.3297
18	TG3, BUN5, NONHDL5	→	LDL5	0.0114	1.0000	5.1239

**Figure 1:**
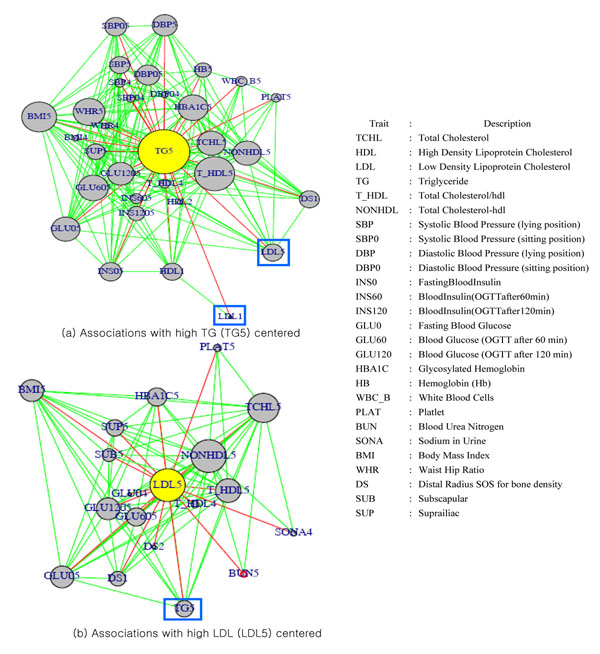
Visualization of phenotypic associations with connected graphs.

Definition of trait names refers to Figure 1.

#### Associations between high TG levels and MS related traits

Association patterns of single traits extracted from 359 rules containing high TG levels were visualized by a connected graph (Figure 1). High TG trait ( TG5 in Figure 1) connected with peculiar nodes representing the 17 distinctive traits: Bone Mineral Density (BMD) measure, distal radius SOS(DS); Blood components, HB, WBC_B, PLAT, and HBA1C; and metabolic syndrome (MS) (Daskalopoulou, et al., 2006) measures, obesity (BMI, WHR and SUP), lipids (LDL, HDL, TCHL, T_HDL, TG and NONHDL), hypertension (SBP and DBP) and insulin resistance (GLU0, INS0, GLU60 and GLU120), post-challenge insulin(INS60 and INS120). The abbreviation of single traits refers to Figure 1.

Associations between high TG levels and a cluster of 4 common traits (obesity, insulin resistance, hypertension, and hyperlipidemia) related to MS, were consistent with the fact that MS increases T2D and cardiovascular diseases (CVD) [20].

#### Associations between high TG levels and BMD

One of the noteworthy findings is the association between low DS for the measure of BMD and high TG levels. The associations between low DS and a cluster of MS defined by the four common traits i.e. obesity, hypertension, hyperlipidemia and insulin resistance with high glucose levels and dissociation with insulin levels (INS0, INS60, INS120) were in concordance with newly reported work [21] that examined an association between MS and bone health. There are negative relations between low DS associated with high levels of lipids including TG, TCHL and LDL and positive relations between low DS with low levels of HDL [22]. More interesting finding was observed in that low DS are associated with high levels of glucose but not with insulin levels although the association between high glucose levels or insulin resistance with BMD has been inconclusive. In contrast, hyperglycemia is known for a predictor of bone loss and osteoporotic fractures [23]. Our finding can be a suggestive evidence that obesity, hypertension and hyperlipidemia among MS related traits might be associated with osteoporosis.

#### Associations with high LDL

High levels of LDL were shown positive relations with BMI, glucose levels and plasma lipids including TCHL, TG, and NONHDL as well as negative relations with DS. We did not find associations between high LDL levels and insulin levels. Interestingly, highLDL have positive relations with single traits related to renal function such as Blood Urea Nitrogen (BUN) and Sodium in Urine (SONA).

#### Pattern of multivariate phenotype *highLDLhighTG*

Among multiple phenotypic associations with high TG, we considered the phenotypic associations which subdivided samples into feasible sizes of cases and controls for GWAS. We focused on contradictory relationship between high TG levels (TG5 in Figure 1(a)) with low or high levels of LDL (LDL1 in Figure 1(a) and LDL5 in Figure 1(b)). That is, there are positive correlations: between TG and LDL-C and TCHL; between LDL-C and HDL as well as negative correlations: between TG and LDL-C; between HDL and TG, LDL-C and TCHL. Both single traits, high TG levels and high LDL levels, shared common traits (BMI, PLAT, TCHL, and GLU0) associated with themselves.

**Figure 2:**
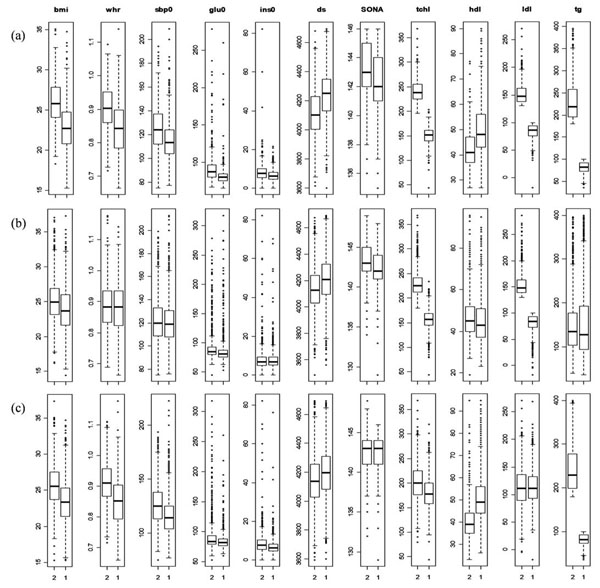
**Distribution of associated traits with multivariate and single traits.** 1 and 2 stand for groups of controls and cases in samples of traits respectively. (a), (b) and (c) stand for *highLDLhighTG*, high LDL and high TG respectively. Out of the 17 associated single traits with high LDL and high TG, 9 single traits were selected for viewing due to keeping image resolution.

Combination of two single traits, high LDL and high TG, introduces multivariate phenotype *highLDLhighTG* which can amplify association strength with correlated single traits by additive effects of the single traits. Out of 17 associated traits, four traits (DS, GLU0, INS0, and SONA) have more power in distinctively classifying samples of *highLDLhighTG* into cases and controls (Figure 2).

The associations between the traits can be substantiated in association rules encoding high TG levels (Rules 1~14) and high LDL-C levels (Rules 15-18). The rules were sorted and selected by their confidences.

As seen from above, there exist complicated associations among single traits. Selection of cases and controls based on single traits without considering those associations may increase confounding effects in samples. Compared with single traits based selection of cases and controls, multivariate based approach can have more power to distinguish cases from controls.

### GWAS results of Plasma lipid levels

**Figure 3:**
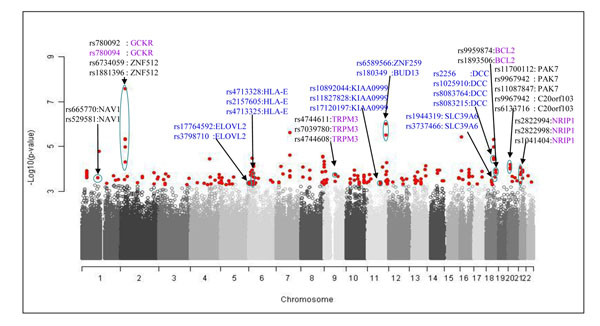
**A manhattan plot for an association test of *highLDLhighTG*.** Gene symbols in purple represents loci identified in previous GWAS of lipids (Kathiresan, et al., 2008). SNPs in blue were pruned.

**Figure 4:**
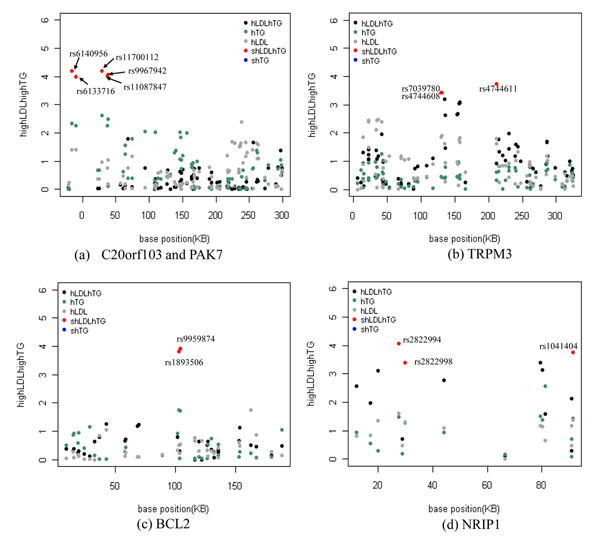
**P-value distributions of association tests for *highLDLhighTG* and single traits highTG and highLDL.** Points in red are significantly identified SNPs in the association test of *highLDLhighTG*. *highLDLhighTG* is presented with *hLDLhTG* and single traits highTG and highLDL are presented in hTG and hLDL respectively.

We identified total 50 variants associated with *highLDLhighTG* and 15 are located in six genes (*PAK7*, *C20orf103*, *NRIP1*, *BCL2*, *TRPM3*, and *NAV1*) (Table 3 and Figure 3). It is interesting to know that rs11700112 of *PAK7* on 20p12.2 is in a missense mutation by substitution of arginine (CGA) by proline (CCA). Clinical association has not yet been found with this variant. It is located within a LD block (530kb) with other four SNPs, of which two (rs6140956 and rs6133716) are in intronic region of *C20orf103*. It is worth to note that *C20orf103* contains a frameshift mutation at rs72238296, which is 755 bases upstream of rs6140956 in the same gene (Table. 3 and Figure 4(a)). The frameshift mutation is known for a cause of a hypercholesterolemia [24].

**Table 3:** Genetic variants associated with *highLDLhighTG*

SNP	Chr	Base position	SNP type	gene	Str- and	Allele(+/-)		Freq (+)	P-value hLDLhTG	OR	mOR_P_^LDL^	mOR_P_^TG^	r^2^
**rs11700112**	20	9495018	nonsyn	PAK7	-	G	C	0.31	6.2×10^-5^	1.44	3.25	1.58	1.00
rs6140956	20	9450080	intronic	C20orf103	+	C	T	0.41	6.3×10^-5^	1.40	2.79	1.85	0.56
rs6133716	20	9455931	intronic	C20orf103	+	A	G	0.40	1.0×10^-4^	1.39	2.58	1.75	0.59
rs9967942	20	9503781	intronic	PAK7	-	C	A	0.34	9.6×10^-5^	1.41	3.24	1.76	0.89
rs11087847	20	9504159	intronic	PAK7	-	T	G	0.31	8.6×10^-5^	1.43	3.02	1.59	0.99
**rs2822994**	21	15282928	intronic	NRIP1	-	A	G	0.41	8.7×10^-5^	1.40	2.46	2.59	1.00
rs2822998	21	15285230	intronic	NRIP1	-	C	A	0.41	4.1×10^-4^	1.35	2.09	2.12	0.97
rs1041404	21	15346738	intronic	NRIP1	-	A	G	0.43	1.7×10^-4^	1.37	2.38	2.33	0.64
**rs9959874**	18	59045526	intronic	BCL2	-	A	G	0.15	1.2×10^-4^	1.60	3.40	2.20	1.00
rs1893506	18	59044660	intronic	BCL2	-	G	A	0.15	1.5×10^-4^	1.59	3.31	2.08	1.00
**rs4744611**	9	72551280	intronic	TRPM3	-	G	A	0.49	1.8×10^-4^	1.36	1.93	3.14	1.00
rs7039780	9	72469777	intronic	TRPM3	-	G	A	0.50	3.7×10^-4^	1.34	1.57	2.98	0.90
rs4744608	9	72470797	intronic	TRPM3	-	G	C	0.50	3.7×10^-4^	1.34	1.52	2.99	0.90
**rs665770**	1	200014747	intronic	NAV1	+	A	G	0.38	2.0×10^-4^	1.38	2.65	1.54	1.00
rs529581	1	200016143	intronic	NAV1	+	C	G	0.37	2.0×10^-4^	1.38	2.59	1.59	0.99
rs693	2	21085700	coding	APOB	-	A	G	0.07	7.3×10^-4^	1.83	0.04	2.21	-

SNP rs693 reported in a previous study (Kathiresan, et al, 2008) for associations between high TG and high LDL. The rs693 was pruned since its effect was stronger in single traits high LDL than multivariate *highLDLhighTG* with borderline significance.

**Figure 5:**
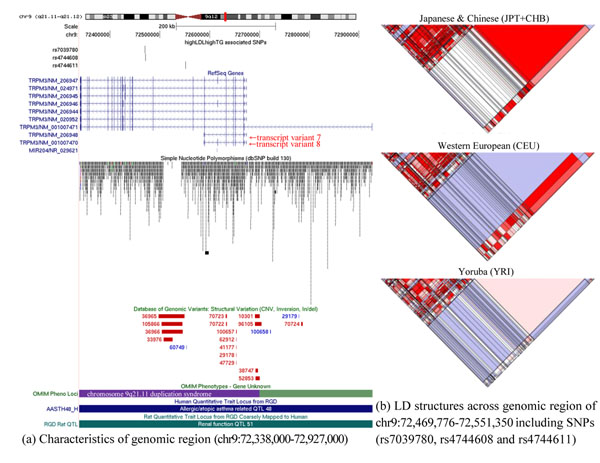
Genomic features for LD structures in HapMap populations.

A strong LD block (81kb length) with high r^2^ values (r^2^ ≥ 0.90) detected across three SNPs (rs4744611, rs7039780 and rs4744608) (Figure 5(b)) of TRPM3 on chromosome 9 (9q21.11-q21.12) that is relatively close to regions linked to coronary artery disease [25]. Among nine splice variants of *TRPM3*, splice variants 7 and 8 do not include the three SNPs identified (Figure 5(a)). This observation suggests that SNPs can make different functional effects on splice variants. Although no firm genetic linkage to disease has been established and not much report on the properties of *TRPM3*, functional activity of TRPM3 is relevant to contractile and proliferating vascular smooth muscle cells. Recent work [26] investigated the relevance and regulation of *TRPM3* in vascular biology and showed that elevated cholesterol can act as a negative regulator of TRPM3.

Two SNPs of *BCL2* gene on chromosome 18 (18q21.33) were identified. *BCL2*, which is involved in a number of cancers including melanoma, breast carcinomas and etc., was recognized as important modulators of cardiac myocyte apoptosis. A distinct support for relevance of *BCL2* to cardio vascular disease (CVD) was reported by recent finding [27] that PPARγ protected cardiac myocytes from oxidative stress and apoptosis through upregulating *BCL2* expression.

*NRIP1* was reported to have an association with HDL [28]. Recent studies identified a hepatocyte specific role for *NRIP1* as a cofactor for *LXR* in different ways, namely serving as a coactivator in lipogenesis and as a corepressor in gluconeogenesis [29]. *NAV1* on chromosome 1q32.1, a human homolog of a *C. elegans* gene, unc-53, is expressed in adult heart and the developing brain. Clinical association has not been established with it. Our results warrant that variants associated with *highLDLhighTG* should be evaluated for further study.

It is important to emphasize that LD structures for the six genes across three populations (YRI, CEU, JPT+CHB) are distinct. The pattern of the strongest LD was observed in JPT+CHB among the three. Whereas, the weak pattern of LD was appeared to be in CEU (see Additional file 4).

### *In silico* replication

*In silico* replication analysis was conducted for the 15 SNPs in two regional subcohorts as well as gender groups (Table 4). Nine of 15 SNPs associated with *highLDLhighTG* were well reproducible in regional subcohorts (P < 0.05), while p-values of six SNPs (p ≥ 0.05) were on the borderline statistical significance. Five SNPs in *NIRP1* (rs2822994, rs2822998 and rs1041404) and *NAVI* (rs665770 and rs529581) were more reproducible in both regional subcohorts and gender groups.

**Table 4:** Replication of GWAS of *highLDLhighTG*

SNP	Ansung			Ansan			Combined	Male			Female			Combined
	
	LDLTG n=205	LDL-C n=919	TG n=936	LDLTG n=340	LDL-C n=1352	TG n=843	LDLTG n=545	LDLTG n=288	LDL-C n=1044	TG n=969	LDLTG n=257	LDL-C n=1227	TG n=810	LDLTG n=545
rs11700112	2.6×10^-2^	0.1818	0.3849	3.1×10^-2^	0.7172	0.0172	2.8×10^-2^	1.8×10^-1^	0.4161	0.6321	1.0×10^-3^	0.0967	0.0072	9.2×10^-2^
rs6140956	8.8×10^-4^	0.1770	0.1017	7.1×10^-2^	0.7749	0.0332	3.6×10^-2^	1.1×10^-1^	0.6353	0.3125	8.4×10^-4^	0.2317	0.0096	5.6×10^-2^
rs6133716	1.8×10^-3^	0.2691	0.0748	9.3×10^-2^	0.7603	0.0701	4.7×10^-2^	1.1×10^-1^	0.9550	0.2231	1.9×10^-3^	0.1757	0.0259	5.8×10^-2^
rs9967942	2.6×10^-2^	0.3476	0.4816	4.1×10^-2^	0.6707	0.0198	3.3×10^-2^	4.1×10^-1^	0.2925	0.8325	6.9×10^-4^	0.1545	0.0072	2.0×10^-1^
rs11087847	1.9×10^-2^	0.1531	0.3200	3.5×10^-2^	0.7218	0.0151	2.7×10^-2^	1.5×10^-1^	0.4459	0.5424	1.1×10^-3^	0.0750	0.0062	7.4×10^-2^
rs2822994	1.1×10^-1^	0.1582	0.2536	2.9×10^-5^	0.0158	0.0019	5.4×10^-2^	1.5×10^-2^	0.4400	0.0060	2.4×10^-4^	0.0045	0.1282	7.9×10^-3^
rs2822998	1.2×10^-1^	0.1985	0.2456	7.4×10^-5^	0.0172	0.0043	5.8×10^-2^	3.2×10^-2^	0.4875	0.0084	2.9×10^-4^	0.0101	0.1361	1.6×10^-2^
rs1041404	1.6×10^-3^	0.0668	0.0357	3.1×10^-3^	0.0274	0.0338	2.4×10^-3^	7.8×10^-3^	0.3843	0.0049	4.4×10^-4^	0.0094	0.0939	4.1×10^-3^
rs9959874	1.2×10^-1^	0.4953	0.1942	1.1×10^-3^	0.0540	0.2536	5.9×10^-2^	4.3×10^-3^	0.4620	0.0720	1.8×10^-1^	0.2007	0.9606	9.3×10^-2^
rs1893506	1.2×10^-1^	0.4953	0.1942	1.3×10^-3^	0.0584	0.2616	5.9×10^-2^	5.3×10^-3^	0.4620	0.0771	1.8×10^-1^	0.2018	0.9797	9.4×10^-2^
rs4744611	1.7×10^-2^	0.9652	0.0078	4.3×10^-2^	0.0090	0.8933	3.0×10^-2^	1.1×10^-3^	0.0113	0.1925	1.8×10^-1^	0.6221	0.4880	9.1×10^-2^
rs7039780	7.8×10^-3^	0.6334	0.0107	1.4×10^-1^	0.0238	0.7387	7.6×10^-2^	2.2×10^-3^	0.0080	0.2893	2.2×10^-1^	0.6317	0.4947	1.1×10^-1^
rs4744608	7.8×10^-3^	0.5846	0.0122	1.6×10^-1^	0.0253	0.6955	8.2×10^-2^	1.8×10^-3^	0.0074	0.2650	2.6×10^-1^	0.6317	0.5936	1.3×10^-1^
rs665770	2.3×10^-2^	0.2715	0.3219	4.7×10^-2^	0.0747	0.3156	3.5×10^-2^	1.6×10^-2^	0.5529	0.1529	8.1×10^-2^	0.0321	0.6182	4.9×10^-2^
rs529581	2.2×10^-2^	0.2498	0.3413	4.5×10^-2^	0.0524	0.3337	3.4×10^-2^	2.4×10^-2^	0.5012	0.1932	6.2×10^-2^	0.0260	0.5976	4.3×10^-2^

rs693	8.0×10^-3^	0.0889	0.2148	1.1×10^-2^	0.0045	0.0396	9.6×10^-3^	2.1×10^-3^	0.0373	0.0147	2.8×10^-2^	0.0056	0.6146	1.5×10^-2^

n represents number of cases.

Reproducibility of gender difference in the 15 SNPs were as follows: *PAK7* and *NRIP1* were more effective in women; *BCL2*, *TRPM3* and *NAV1* were more reproducible in men. *highLDLhighTG* was more detectable in women than man (χ^2^ ≥44.9, p-value = 2.05 × 10 ^-11^). *PAK7* and *NRIP1* may lead to the gender specific susceptibility in concordance with previous work [30] reporting more gender-specific effects for CVD in women than men.

### Comparison of general GWAS

**Figure 6:**
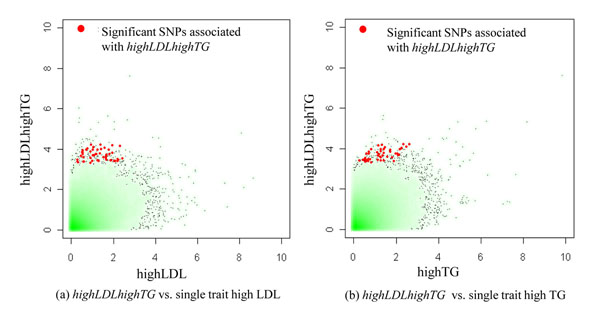
Scatter plots for p-value for a multivariate trait versus single traits.

Overall distribution of p-values for an association test appeared to be less significant than those for general GWAS. On the other hand, the p-values of significant SNPs identified for multivariate phenotype *highLDLhighTG* were apparently more significant than those for single traits highLDL and highTG (Figure 6). It is noteworthy that effect sizes of the significant SNPs which ranged between modest (odds ratios=1.38-1.60) and intermediate effect sizes were comparable to those for the general GWAS ranged from low to modest ones.

### Pleiotropic patterns of quantitative trait loci

Pleiotropic patterns can be more precisely observed in quantitative trait loci (QTLs) or LD blocks than at SNPs. We examined QTLs and their associated phenotypes for the six genes identified using Phenotype and Disease Association track group in UCSC genome browser. The QTLs and their associated phenotypes were extracted from rat and mouse QTLs from RAT DB and MGI (Mouse Genome Information) (Table 5).

**Table 5:** Phenotypes associated with QTLs mapped to 6 genes identified

Gene(s) (chromosome band)	OMIM phenotype (OMIM number)	Phenotypes for rat QTL from RGD	Phenotypes for mouse QTL from MGI
PAK7, C20orf103 (20p12.2)	Body mass index(608559), Atopic dermatitis(605804), Systemic lupus erythematosus(610065), Glaucoma(608696), Alzheimer disease(607116)	Blood pressure, Body weight, Cardiac mass, Stress response, Non-insulin dependent diabetes mellitu, Renal disease susceptibility, Thymus enlargement suppressive	Blood glucose level, Type 2 diabetes mellitus, Bone mineral density, Crescentic glomerulonephritis, Modifier of retinal degeneration

NRIP1 (21q11.2)	Myeloproliferative syndrome(159595) Narcolepsy(609039), Autism(610838)	Testicular tumor resistance	

BCL2 (18q21.33)	Orthostatic hypotensive disorder (143850), Insulin-dependent diabetes mellitus(601941), Amyotrophic lateral sclerosis(606640)	Blood pressure, Cardiac cell morphology, Insulin dependent diabetes mellitus, Renal function	Bone mineral density

TRPM3 (9q21.11 -9q21.13)	Hematocrit/hemoglobin quantitative trait(609320), Cataract(605749), Pelvic organ prolapse(613088), Deafness (chromosome 9q21.11 duplication syndrome)(613558), Epilepsy(611631), paraplegia(607152), Otosclerosis(612096), Spastic Amyotrophic lateral sclerosis(105550)	Blood pressure, Body weight, Heart rate, Stress response, Cardiac mass, Glucose level, Lipid level, Renal function, Kidney mass, Renin concentration, Thyroid stimulating hormone level, Abnormal inflammatory response, Hepatocarcinoma susceptibility,	Atherosclerosis, Bone mechanical trait, Autoimmune aoritis, Cataract severity

NAV1 (1q32.1)	Inflammatory bowel disease(612381), Pseudohypoaldosteronism(145260), Parkinson disease(613164), Glomerulopathy(601894)	Blood pressure, Cardiac mass, Stress response, Renal function, Thymus enlargement, Abnormal inflammatory response	Crescentic glomerulonephritis, Bone mineral density

Phenotypes associated with QTLs were extracted from Phenotype and Disease Association tracks in UCSC genome browser. Phenotypes for OMIM, rat QTL, mouse QTL corresponded to OMIM phenotype loci, RGD RAT QTL and MGI Mouse QTL tracks respectively.

The six genes except *NRIP1* share QTLs commonly associated traits such as BMD and a cluster of common traits defining MS. Those common traits in MS shared by the six genes are blood pressure, non-insulin dependent diabetes mellitus, renal function, cardiac mass, and body weight. The phenotypic associations of high TG and high LDL levels with low BMD examined through rat and mouse QTL associations except *NRIP1* have mapped in the regions of QTLs associated with BMD. Furthermore it can be more support that *TRPM3* was mapped to OMIM phenotypes such as osteosclerosis hardening bones, epilepsy, amyotrophic lateral sclerosis (ALS), of which association with CVD was reported in a recent work [31]. Different genetic markers share the same or similar OMIM phenotypes: *BCL2* and *TRPM3* have in common with associating ALS; *PAK7* and *NAV1* have in common with similar phenotypes Alzheimer disease (AD) and Parkinson disease (PD) where a cross-talk between MS and AD was reported [32].

In summary, our results suggest that the genetic markers identified with multivariate phenotype *highLDLhighTG* have phenotypic associations with common traits in MS. The common traits in MS, particularly hyperlipidemia, may be linked to pathogenic associations with osteosclerosis and neurodegenerative disorders including AD and PD influenced by pleiotropic genetic factors. Thus, the genetic markers identified in our work can have pleiotropic effect on MS, BMD and neurodegenerative disorders.

### Gene network analysis using protein-protein interactions

**Figure 7:**
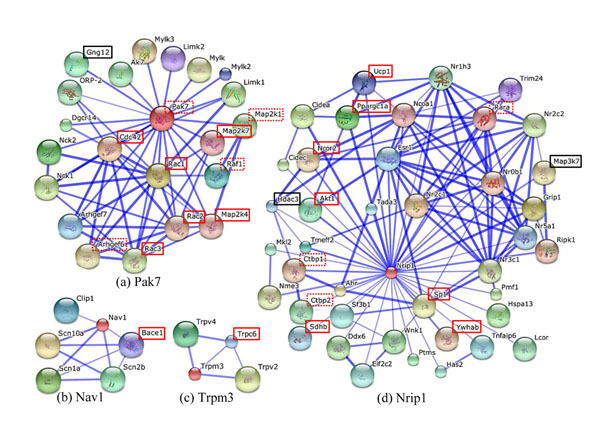
**Gene networks constructed from interacting proteins.** Solid lines in red stand for genes in pathways for AD, PD, HD, and ALS. Gene symbols in black are involved in chemokine, MAPK and Wnt signaling pathways. Dashed lines in red represent genes mapping to pathways in cancer from KEGG DB or specific cancer related pathways annotated by PANTHER and DAVID functional annotations.

We explored possible functional relationships between five of six genes associated with *highLDLhighTG* using STRING, a database of predicted protein-protein interactions (PPI). We obtained 5 different networks of genes interacting with each of five genes by confidence of association evidence (≥ 0.5). Each of the gene networks (Figure 7) was mapped to KEGG pathways and examined pathways in common. Four genes i.e. *BCL2*, *NAV1*, *NIRP1* and *TRPM3* interact with genes (*CASP7*, *BACE1*, *SDHB*, *TRPC6*) in AD and PD pathways, while *BCL2* and *NIRP1* shared Huntington’s disease as well as AD. In particular, three genes i.e. *BCL2*, *PAK7*, and *NIRP1* shared pathways in cancer and other pathways, supporting our hypothesis that multivariate phenotypes have common etiology pathways when they are affected by pleiotropic genetic factors.

## **Conclusions**

We have showed a methodology to identify genetic markers associated with multivariate phenotypes derived from patterns of phenotypic associations discovered by ARM. An application of a large scale mining approach to anthropometric and biochemical traits has not been previously reported.

The patterns of phenotypic associations were visualized with connected graphs which were informative to draw relations between plasma lipid levels with BMD and a cluster of common traits (Obesity, hypertension, insulin resistance) related to MS. These putative patterns of the phenotypic associations were in concordance with the fact that MS increases T2D and CVD [20]. More interesting finding was observed in that BMD was associated with high levels of glucose but not with insulin levels although the association between high glucose levels or insulin resistance with BMD has been inconclusive. We suggest that multiple phenotypic associations between plasma lipid levels with BMD and common traits in MS, be affected by the common genes harbouring pleiotropic effects.

For the identification of pleiotropic genes, we derived multivariate phenotype *highLDLhighTG* from the association patterns of two single traits high LDL and high TG, which subdivided samples into feasible sizes of cases and controls for GWAS. Multivariate phenotype *highLDLhighTG* increased the strength of phenotypic associations with the correlated single traits by additive effect of multiple single traits. Out of 17 traits linked to single traits high LDL and high TG, four traits (DS, GLU0, INS0, and SONA) have more power in distinctively classifying samples of *highLDLhighTG* into cases and controls when the single traits were combined into the multivariate phenotype.

Our approach to GWAS of multivariate *highLDLhighTG* identified 15 SNPs in six genes (*PAK7*, *C20orf103*, *NRIP1*, *BCL2*, *TRPM3*, and *NAV1*). While p-values (9.6 × 10^-5^ ≤ P ≤ 1.2 × 10^-4^) of genetic variants in the six genes were less significant than those identified in general GWAS due to limited sizes of the sample those genes were not detectable within univariate framework of the GWAS. Effect sizes (odds raios=1.34-1.60) of the variants ranged between modest and intermediate sizes were comparable to those in the general GWAS. Relevance of the six genes to CVD in MS was apparently explained in previous work.

According to analysis based on rat and mouse QTL DB, our results suggest that the six genes were mapped to QTLs associated with common traits related to MS, supporting that *highLDLhighTG* represents one of pleiotropic patterns related to MS and the six genes associated with it can influence the pleiotropic effects on MS. In particular, we showed possibility that hyperlipidemia may be linked to pathogenic associations with osteoporosis and neurodegenerative disorders including AD and PD by incorporating associations of OMIM phenotypes with PPI networks for the six genes. We have found that four genes i.e. *BCL2*, *NAV1*, *NIRP1* and *TRPM3* may share AD and PD pathways where interacting genes with them are involved. Three genes i.e. *BCL2*, *PAK7*, and *NIRP1* also share common pathways in cancer.

Clinical association studies for 4 genes i.e. *PAK7*, *C20orf103*, *TRPM3* and *NAV1* have not yet been found although characteristics of genomic features for the four genes are also suggestive to investigate further study. Our work emphasize that multivariate phenotypes based GWAS can identify pleiotropic genes that share common etiology pathways.

## **Authors' contributions**

SHP developed the concept and the method. JYL implemented a module for visualizations of association rules with networks and participated in drafting the paper. SK interpreted the results for the point of view of biology. SHP drafted the paper and JYL and SK were contributed to finalize the draft.

## **Competing interests**

The authors declare that they have no competing interests.

## Supplementary Material

Additional file 1 **Description of traits.** A table shows 52 traits and their description and measurement. Click here for file

Additional file 2 **Basic characteristics for traits.** Baseline characteristics according to means and standard deviations. Click here for file

Additional file 3 **Association rules encoding high TG and high LDL levels.** Representative association rules encoding high TG and high LDL. Click here for file

Additional file 4 Features for genomic regions and LD structures. Click here for file
