# Supplementary material for: A methodology for multivariate phenotype-based genome-wide association studies to mine pleiotropic genes
Source: BMC Syst Biol. 2011 Dec 14;5(Suppl 2):S13. doi: 10.1186/1752-0509-5-S2-S13 (PMC3287479; doi:10.1186/1752-0509-5-S2-S13)

C20orf103- chr20:9,443,271-9,459,171

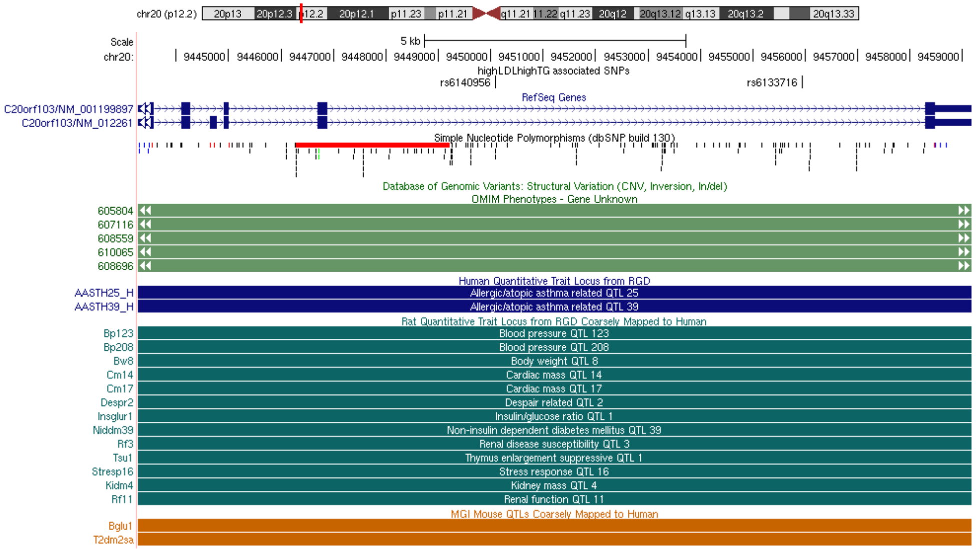

PAK7- chr20:9,466,037-9,767,687

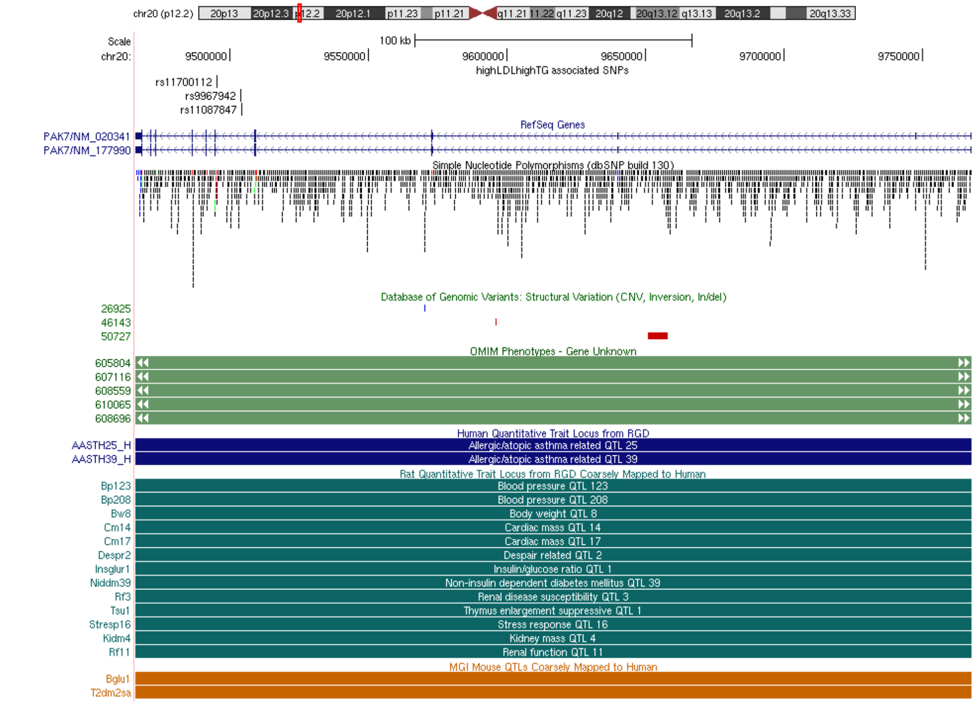

LD for 5SNPs on chr20:9,449,800-9,504,500

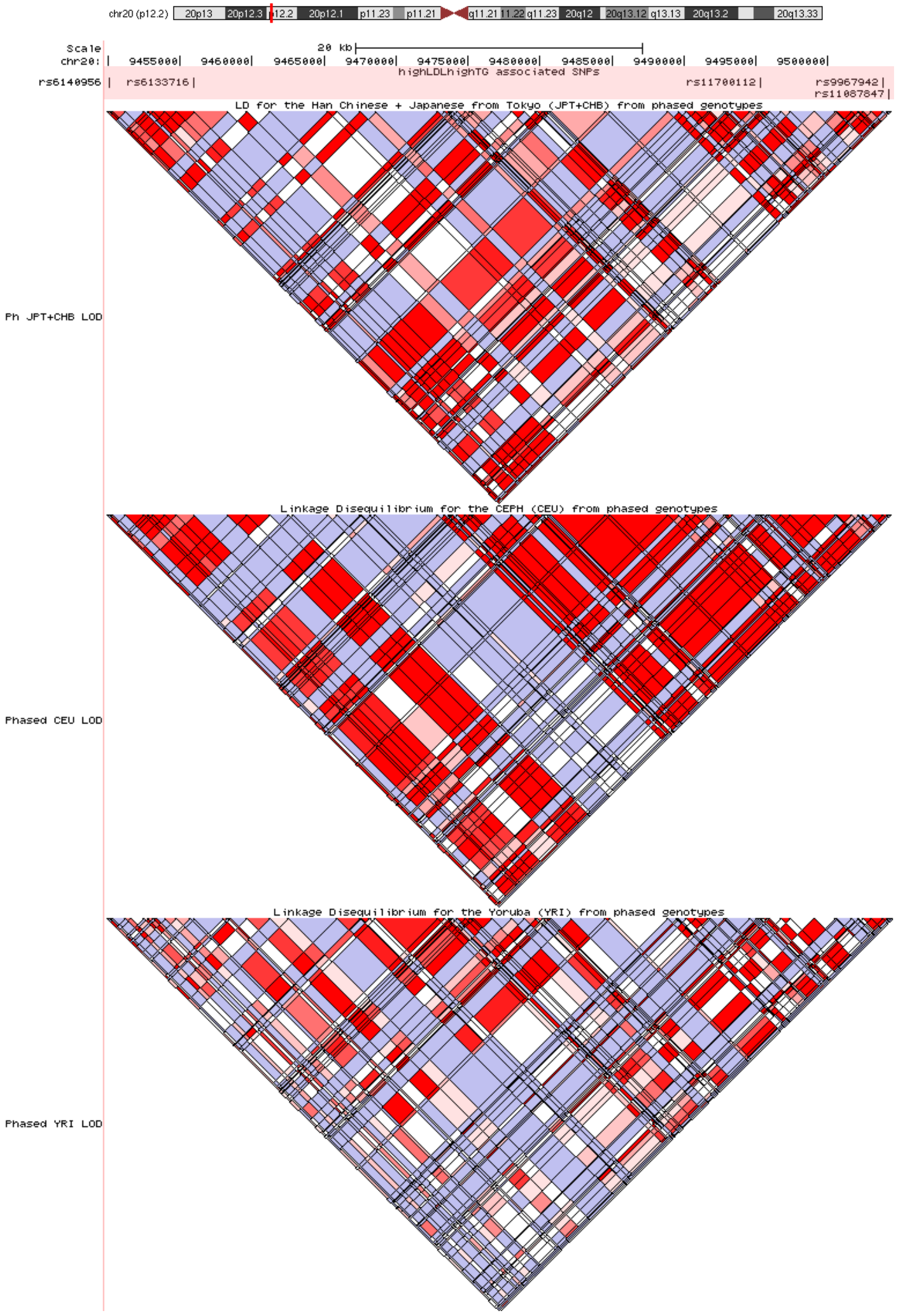

NRIP1- chr21:15,255,427-15,359,000

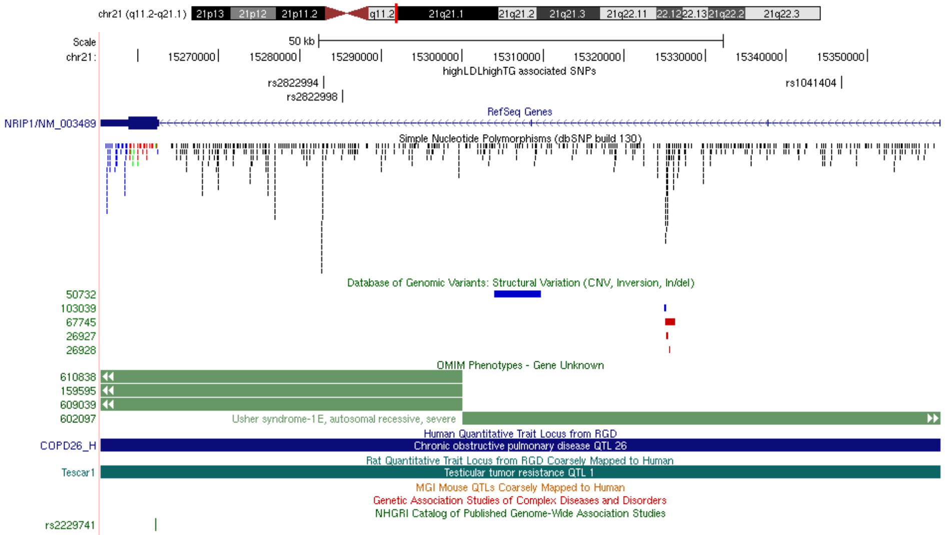

LD for 3SNPs on chr21:15,282,928-15,346,780

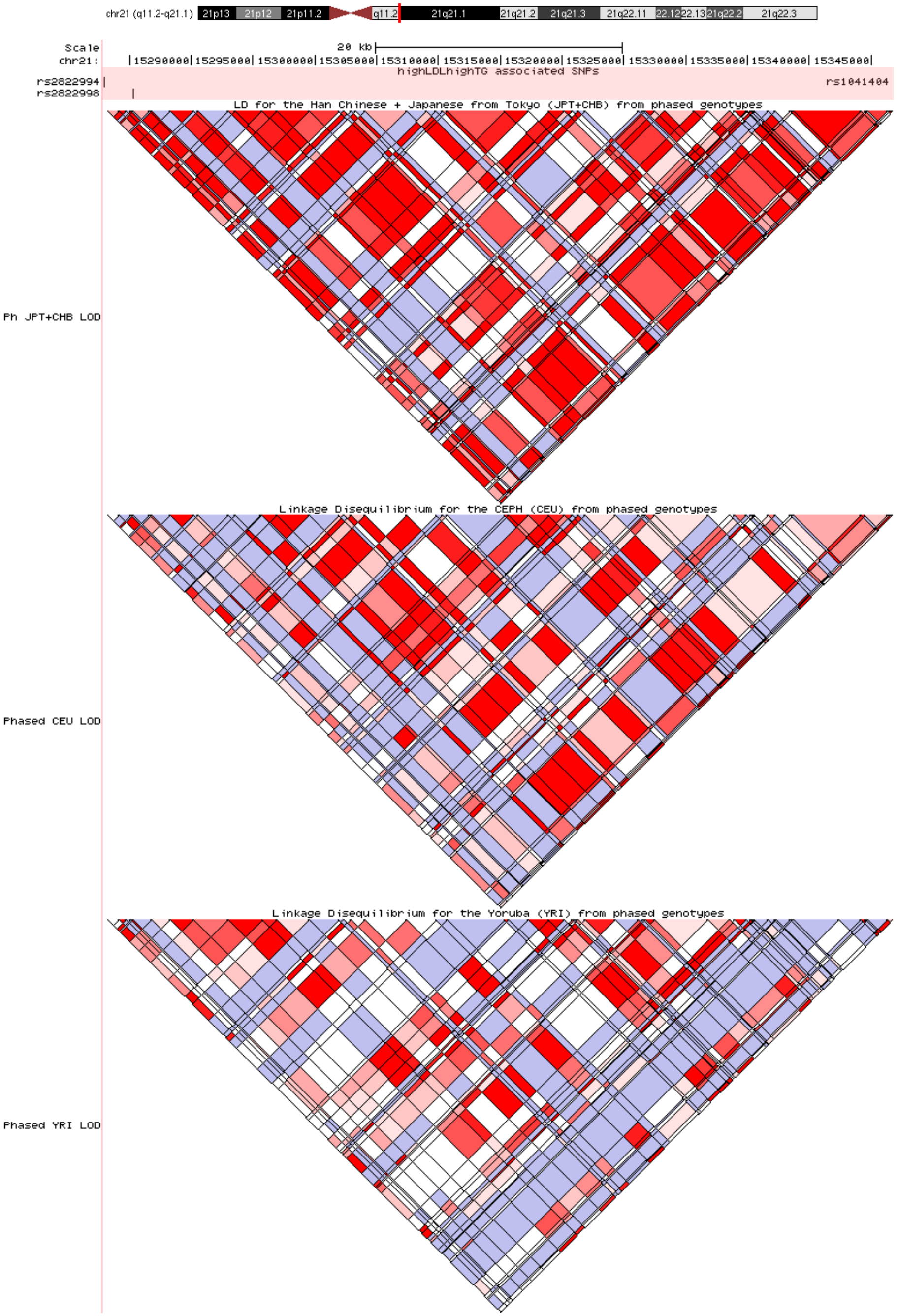

BCL2- chr18:58,941,559-59,137,600

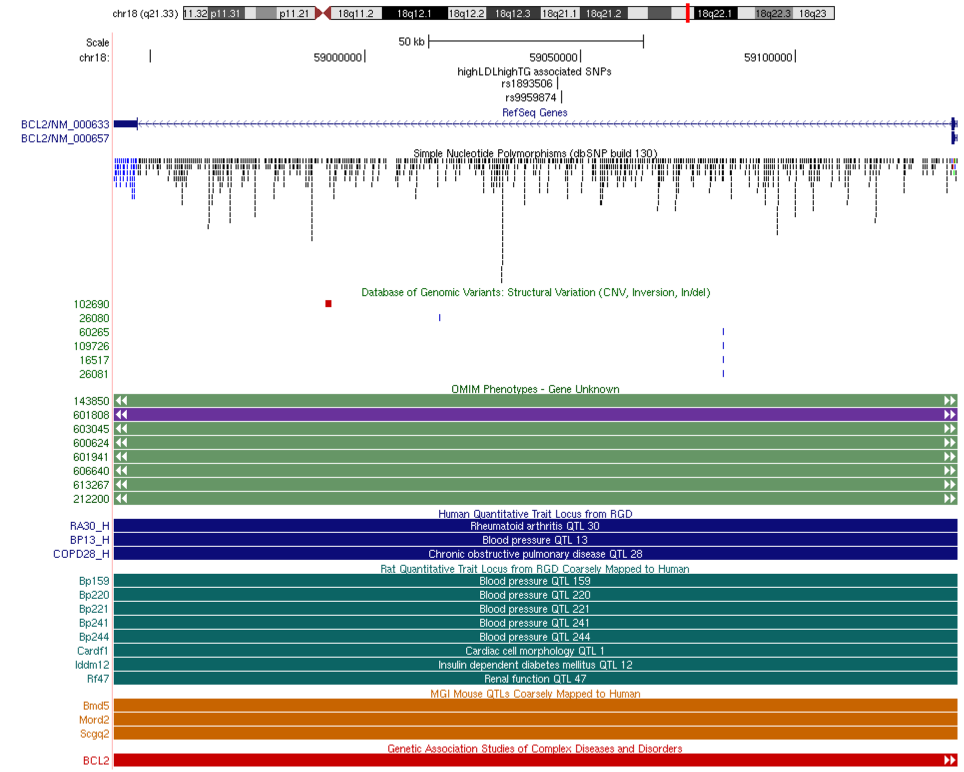

LD for 2SNPs on chr18:59,015,832-59,074,354

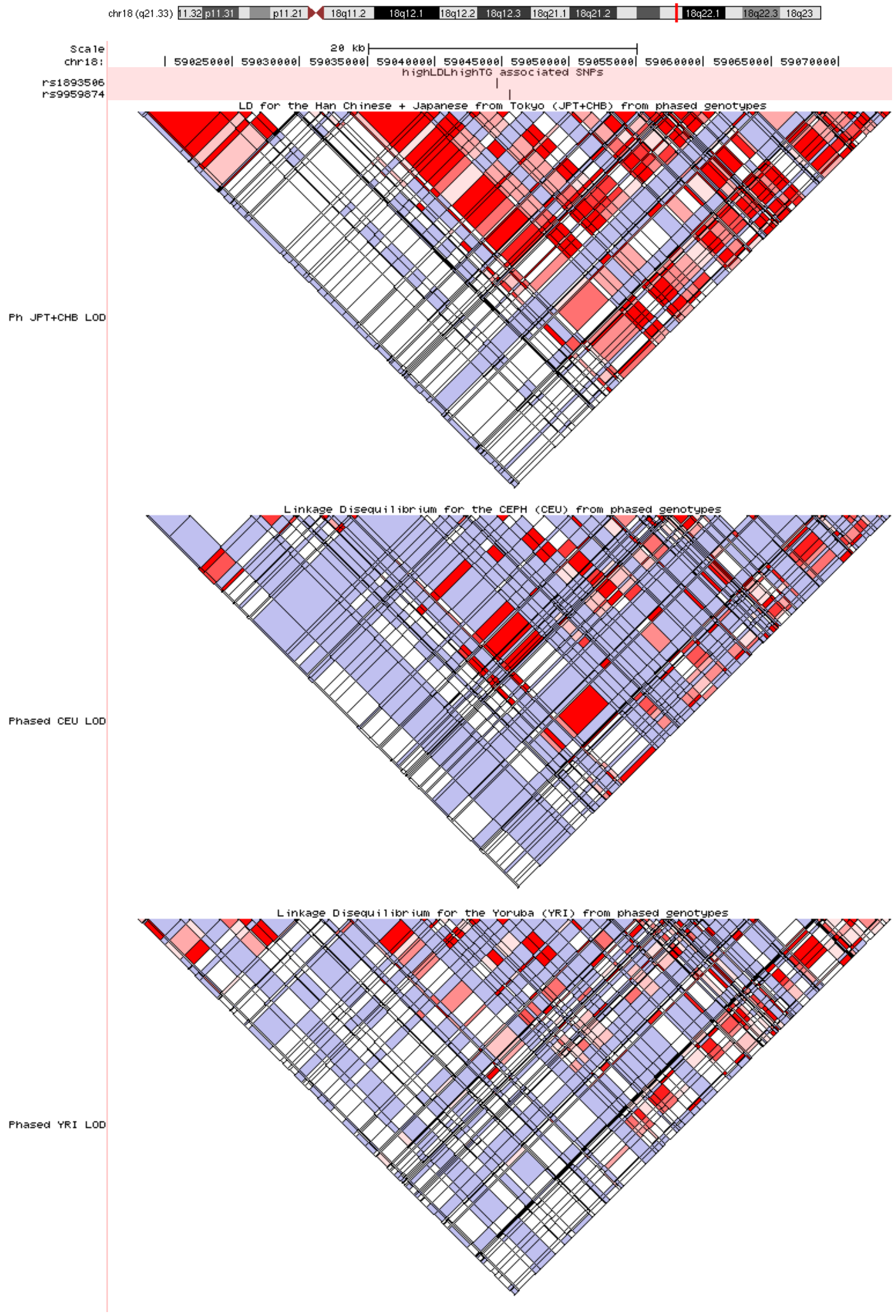

TRPM3- chr9:72,339,786-72,926,334

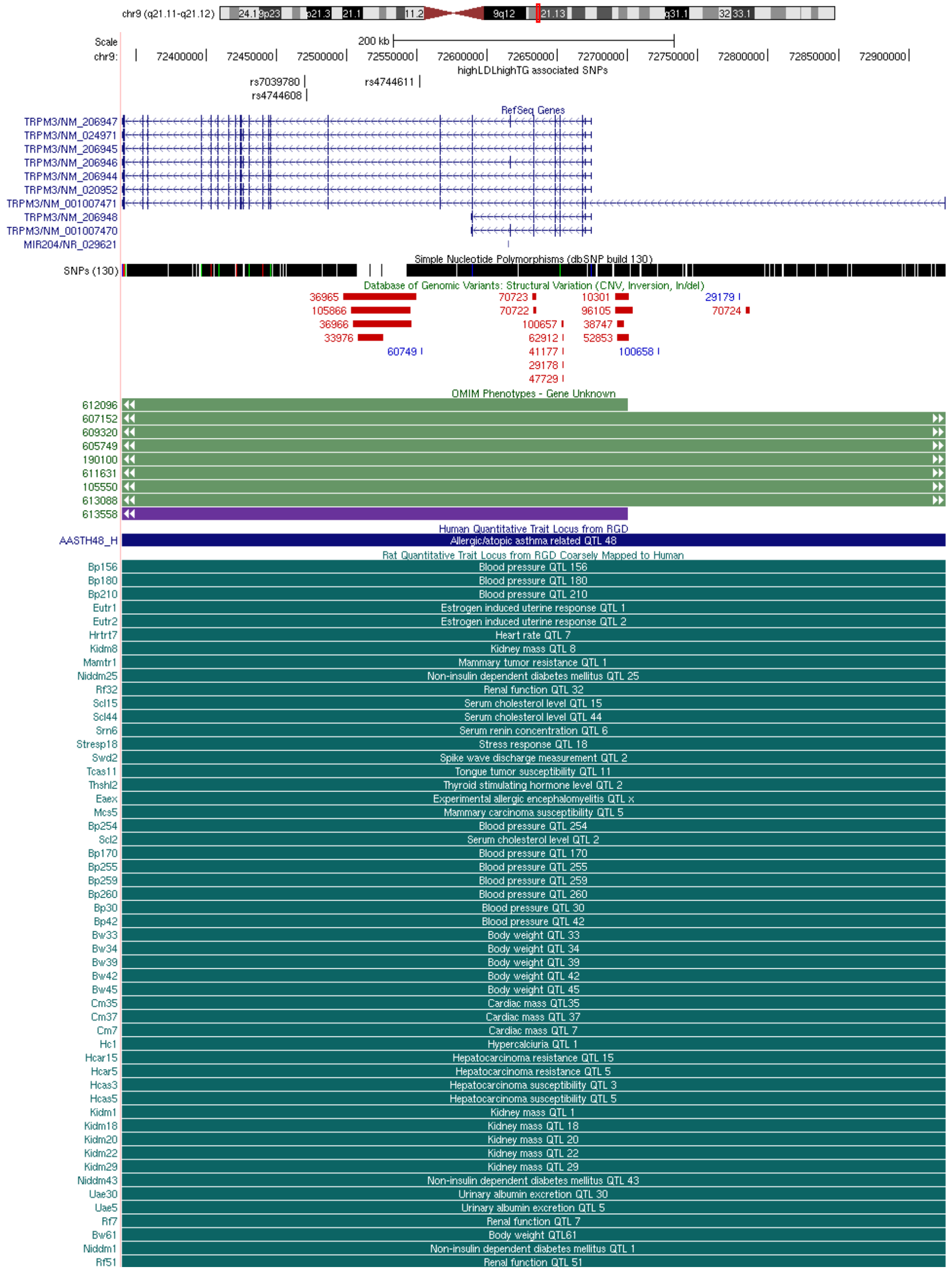

LD for 3SNPs on chr9:72,469,777-72,551,400

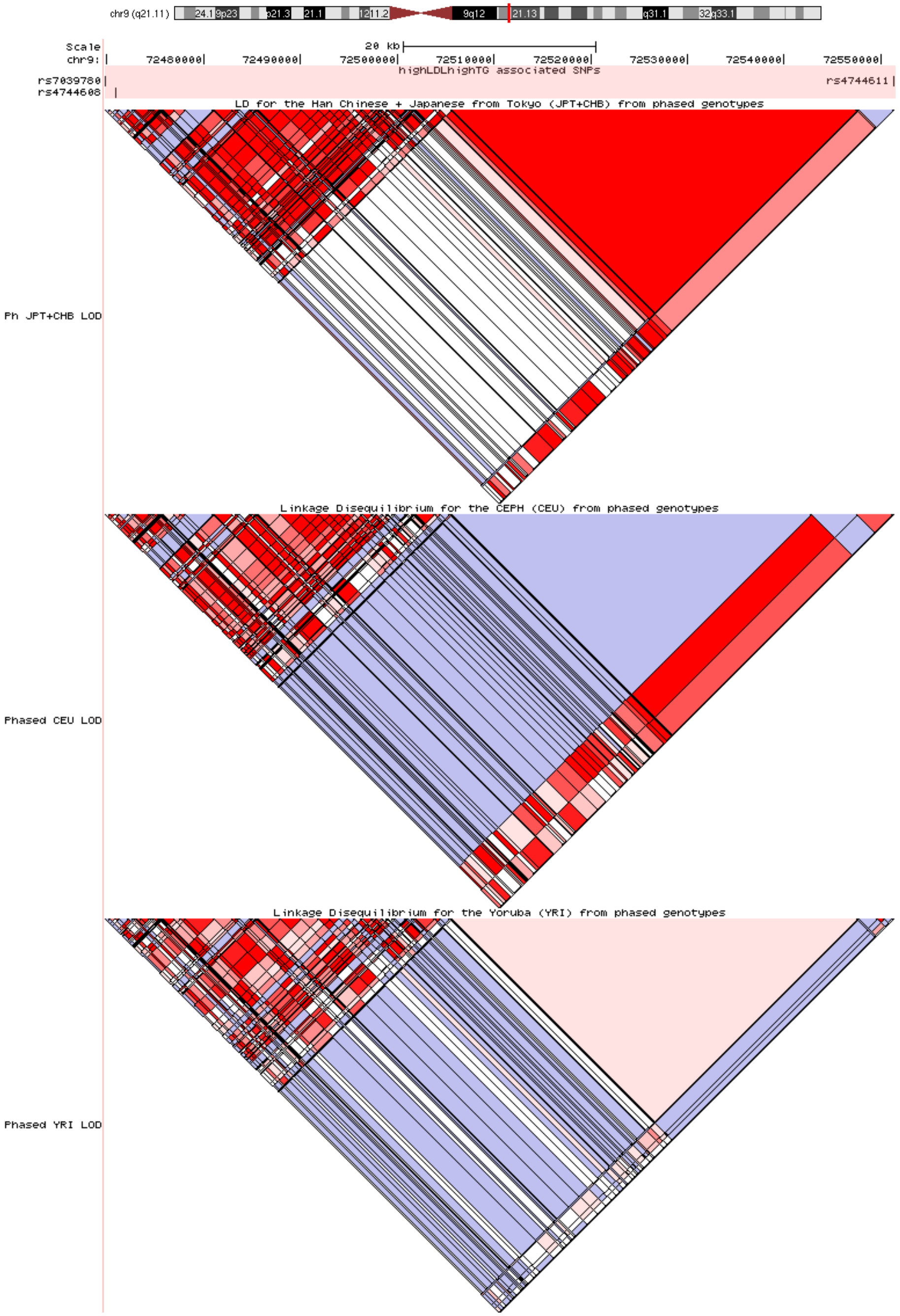

NAV1- chr1:199,884,073-200,062,850

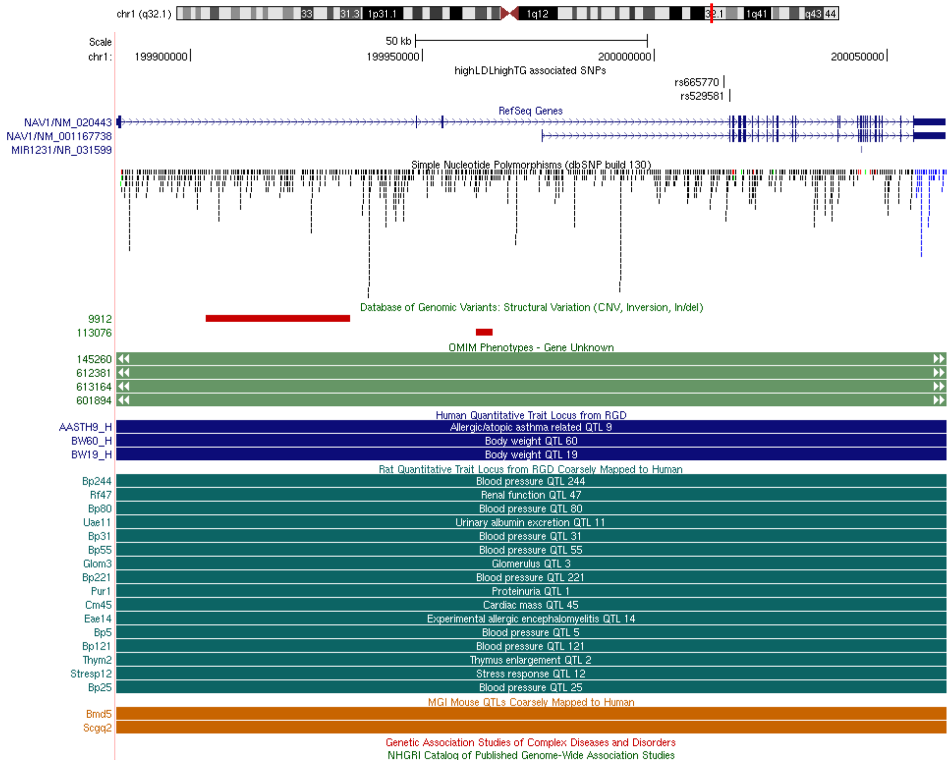

LD for 2SNPs on chr1:199,980,083-200,050,807

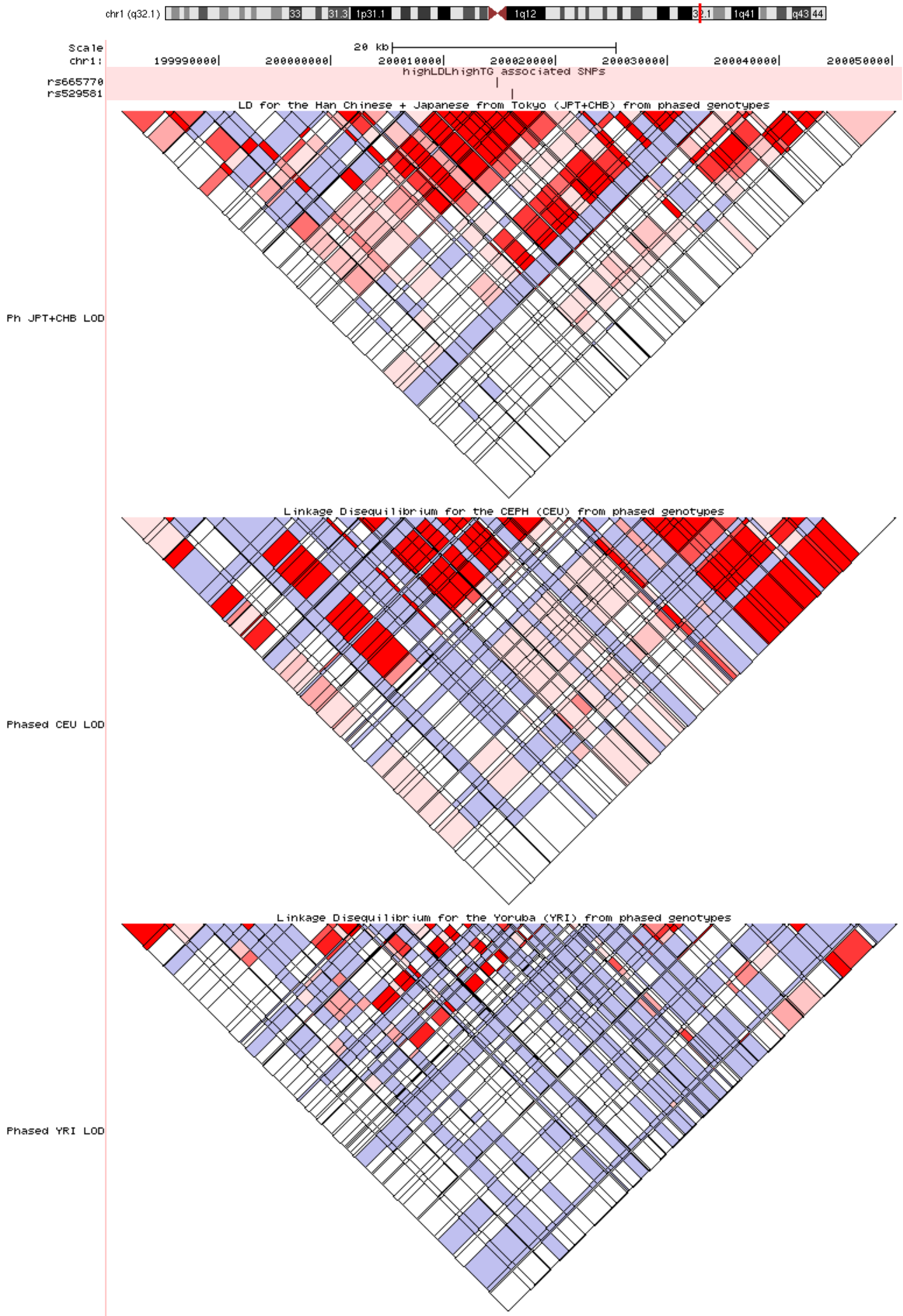

TRPM3- chr9:72,339,786-72,926,334

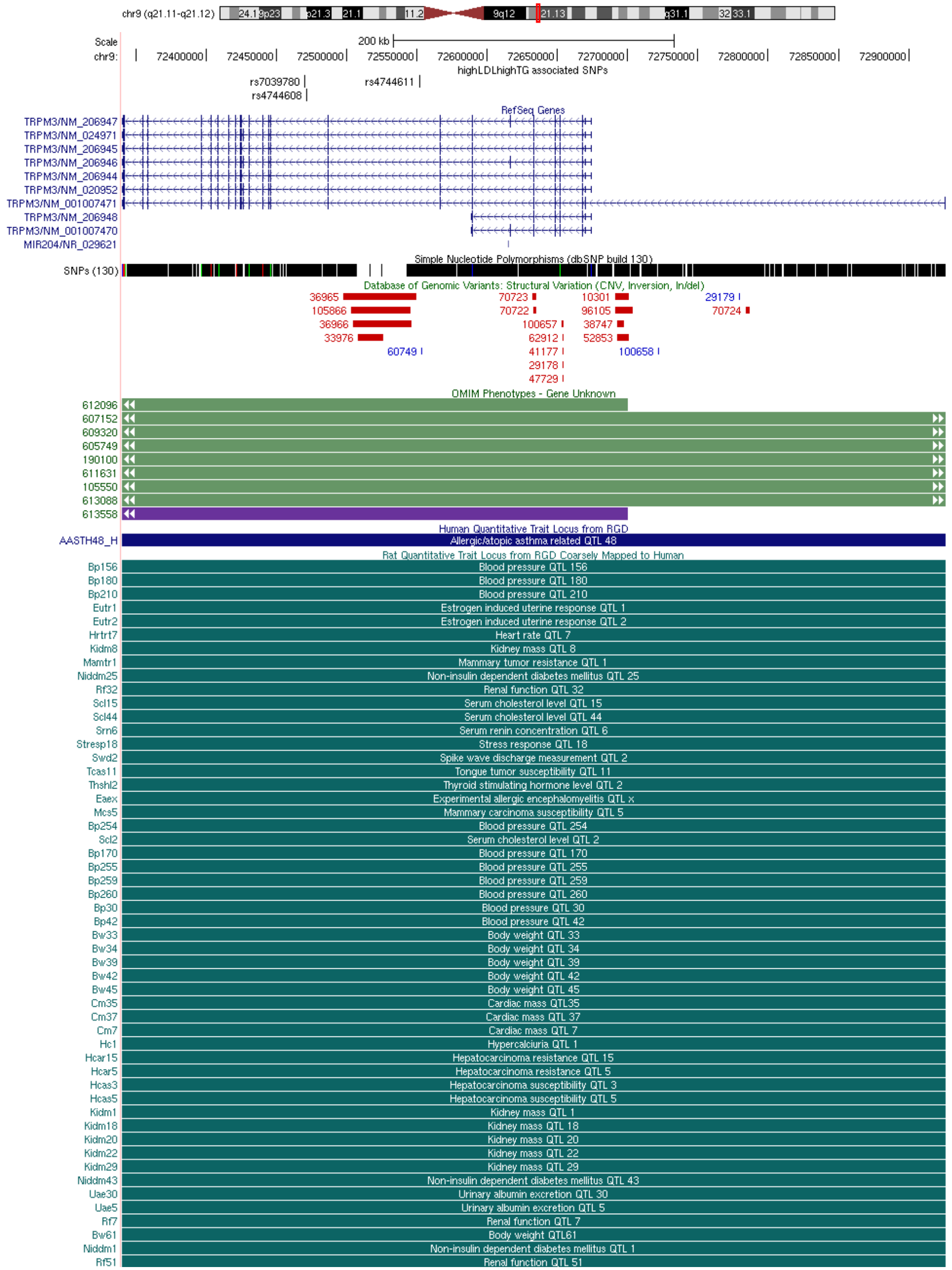

LD for 3SNPs on chr9:72,469,777-72,551,400

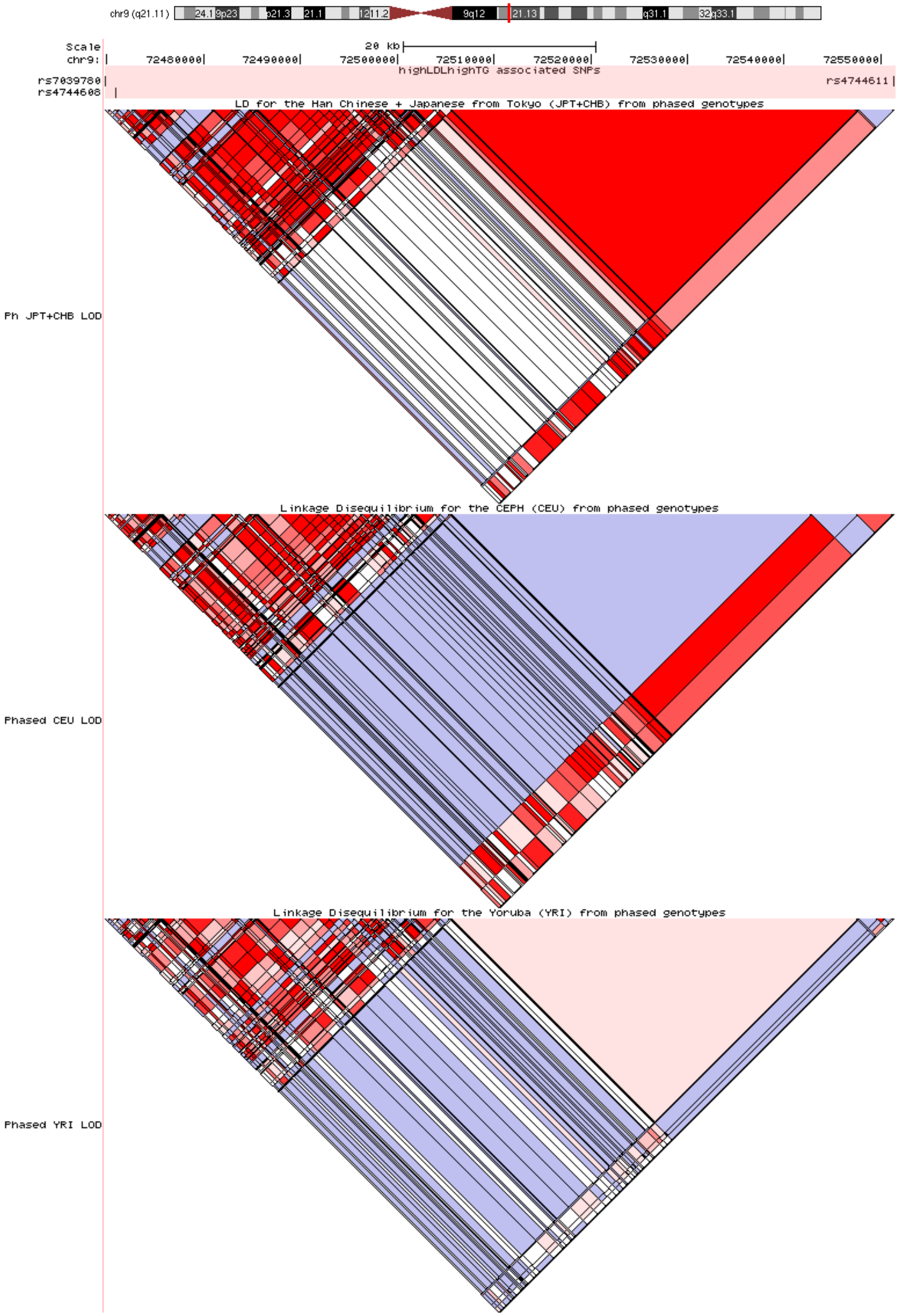

Supplement: Additional file 4 — Features for genomic regions and LD structures. [file 1752-0509-5-S2-S13-S4.pdf]
